# Association between rosacea and increased cardiovascular risk factors: A case-control study using the DataDerm database

**DOI:** 10.1016/j.jdin.2024.08.014

**Published:** 2024-09-16

**Authors:** Divija Sharma, Angel D. Pagan, Stella A. Caldas, Joel Correa da Rosa, Arik Aninos, Benjamin Ungar

**Affiliations:** a Department of Dermatology, Icahn School of Medicine at Mount Sinai, New York, New York; b Ponce Health Sciences University School of Medicine, Ponce, Puerto Rico; c George Washington University School of Medicine, Washington, District of Columbia; d American Academy of Dermatology, Rosemont, Illinois

**Keywords:** cardiovascular disease, hypertension, ischemic heart disease, metabolic disorder, rosacea, stroke

*To the Editor:* Rosacea is a chronic inflammatory skin disease characterized by centrofacial erythema, swelling, and the appearance of papules and pustules. Recent studies suggest a link between rosacea and cardiovascular disease (CVD)^1^^,^^2^; however, this association remains equivocal due to inconsistencies in previous literature.^3^ CVD remains a leading cause of death, necessitating comprehensive strategies for early detection and management. This study aims to add additional analysis on the potential relationship using DataDerm, the American Academy of Dermatology’s clinical registry.

A nested case-control study was conducted among adult (>18 years) rosacea patients using International Classification of Diseases-10 (ICD-10) codes L71.0, L71.8, L71.9 on at least two occasions. Cardiovascular-related conditions were identified using grouped ICD-10 codes (Supplementary Table I, available via Mendeley at https://data.mendeley.com/datasets/f8h65mr549/1): (1) hypertensive disorders (HTN); (2) endocrine, nutritional, and metabolic disorders (ENM); (3) peripheral vascular disease (PVD); (4) ischemic heart disease (IHD); and (5) other heart disease (oHD). Rosacea patients were matched 1:4 to controls using propensity scores based on age, gender, and race/ethnicity. Statistical analysis was performed using conditional logistic regression to estimate odds ratios (ORs) and confidence intervals (CIs).

We identified 112,577 rosacea patients and 450,308 controls (Table I). Rosacea patients were slightly less likely to have HTN than controls, though this only approached statistical significance (OR: 0.85; 95% CI: 0.71-1.02; *P* = .074). Rosacea patients were significantly more likely to have ENM (OR: 1.50; 95% CI = 1.28-1.76; *P* < .001) and PVD (OR: 2.16; 95% CI: 1.77-2.61; *P* < .001). No significant differences were found in IHD (OR: 1.11; 95% CI = 0.41-2.99; *P* = .835) or oHD (OR: 1.24; 95% CI = 0.73-2.11; *P* = .423; Table II).

**Table I:** Demographic table of rosacea patients versus matched controls 1:4

	Total *N* = 562,895	Rosacea *N* = 112,577	Controls *N* = 450,308
Age	56.6 (±16.8)	57.3 (±15.6)	56.4 (±17.1)
Gender			
Female	406,828 (72.27%)	81,362 (72.27%)	325,466 (72.28%)
Male	156,057 (27.72%)	31,215 (27.73%)	124,842 (27.72%)
Race			
African American	7336 (1.30%)	1465 (1.30%)	5871 (1.30%)
American Indian or Alaskan Native	224 (0.04%)	38 (0.03%)	186 (0.04%)
Asian	4934 (0.88%)	978 (0.87%)	3956 (0.88%)
Hispanic/Latino	2104 (0.37%)	377 (0.33%)	1727 (0.38%)
Multiracial	230 (0.04%)	40 (0.04%)	190 (0.04%)
Native Hawaiian or Pacific Islander	116 (0.02%)	33 (0.03%)	83 (0.02%)
Other	7121 (1.27%)	1476 (1.31%)	5645 (1.25%)
White	540,820 (96.08%)	108,170 (96.09%)	432,650 (96.08%)
Ethnicity			
Hispanic/Latino	12,885 (2.29%)	2628 (2.33%)	10,257 (2.28%)
Non-Hispanic/Latino	550,000 (97.71%)	109,949 (97.67%)	440,051 (97.72%)

**Table II:** Logistic regression outcomes of grouped variables of rosacea patients versus matched controls 1:4

Grouped cohort	Participants with rosacea *N* = 112,577	Participants without rosacea *N* = 450,308	Odds ratio (OR)	Confidence interval (CI)	*P* value
Hypertension (HTN)	155 (0.14%)	726 (0.16%)	0.85	0.72, 1.02	.074
Endocrine, nutritional, metabolic disorders (ENM)	208 (0.18%)	554 (0.12%)	1.50	1.28, 1.76	<.001
Peripheral vascular disease (PVD)	146 (0.13%)	270 (0.06%)	2.16	1.77, 2.65	<.001
Ischemic heart disease (IHD)	5 (0.004%)	18 (0.004%)	1.11	0.41, 2.99	.835
Other heart disease (oHD)	18 (0.02%)	58 (0.01%)	1.24	0.73, 2.11	.423

This study highlights a complex relationship between rosacea and CVD; a significantly increased likelihood of ENM and PVD was accompanied by a trend toward decreased HTN, with no signal found for IHD or oHD. The common pathogenic mechanisms and risk factors linking rosacea and CVD may stem from chronic inflammation triggered by immune dysregulation, which is compounded by shared risk factors including smoking, alcohol consumption, and dietary habits.^1^

A major strength of this study is the use of the DataDerm database that primarily includes dermatologists’ clinical notes, reducing the likelihood of rosacea misdiagnosis. In 2020, the database underwent a rigorous audit process involving a manual review of 1098 patient charts from 2018 across eight dermatology practices using four different electronic health record systems. The audit revealed a completeness of 95.3% and an accuracy of 89.8% of dermatologic diagnoses.^4^ According to the 2023 report, DataDerm has become the largest dermatology database, capturing extensive information about patient’s skin disease courses, treatment interventions, and health outcomes.^5^ DataDerm has made significant progress with its collaboration with OM1, the database’s analytics partner, which has provided it with a robust representation of dermatologic care over time.^5^

A notable limitation includes the low rates of cardiovascular diagnoses, likely a product of under-documentation of non-dermatologic diseases in the database. This mismatch also suggests room for improvement in the dermatologic community for documenting medical history more comprehensively. Overall, the results of this study provide additional support for cardiometabolic associations in patients with rosacea.

## Conflicts of interest

Dr Ungar is an employee of Mount Sinai and has received research funds (grants paid to the institution) from Incyte, Rapt Therapeutics, and Pfizer. He is also a consultant for Arcutis Biotherapeutics, Bristol Myers Squibb, Castle Biosciences, Fresenius Kabi, Galderma, Janssen, Lilly, Pfizer, Primus Pharmaceuticals, Sanofi, and UCB. Drs Sharma, Pagan, Caldas, Correa da Rosa, and Aninos have no conflicts of interest to declare.
